# A Universal Isotherm Model to Capture Adsorption Uptake and Energy Distribution of Porous Heterogeneous Surface

**DOI:** 10.1038/s41598-017-11156-6

**Published:** 2017-09-06

**Authors:** Kim Choon Ng, Muhammad Burhan, Muhammad Wakil Shahzad, Azahar Bin Ismail

**Affiliations:** King Abdullah University of Science and Technology, Thuwal, 23955-6900 Saudi Arabia

## Abstract

The adsorbate-adsorbent thermodynamics are complex as it is influenced by the pore size distributions, surface heterogeneity and site energy distribution, as well as the adsorbate properties. Together, these parameters defined the adsorbate uptake forming the state diagrams, known as the adsorption isotherms, when the sorption site energy on the pore surfaces are favorable. The available adsorption models for describing the vapor uptake or isotherms, hitherto, are individually defined to correlate to a certain type of isotherm patterns. There is yet a universal approach in developing these isotherm models. In this paper, we demonstrate that the characteristics of all sorption isotherm types can be succinctly unified by a revised Langmuir model when merged with the concepts of Homotattic Patch Approximation (HPA) and the availability of multiple sets of site energy accompanied by their respective fractional probability factors. The total uptake (q/q*) at assorted pressure ratios (*P/P*_*s*_) are inextricably traced to the manner the site energies are spread, either naturally or engineered by scientists, over and across the heterogeneous surfaces. An insight to the porous heterogeneous surface characteristics, in terms of adsorption site availability has been presented, describing the unique behavior of each isotherm type.

## Introduction

**Figure 1:**
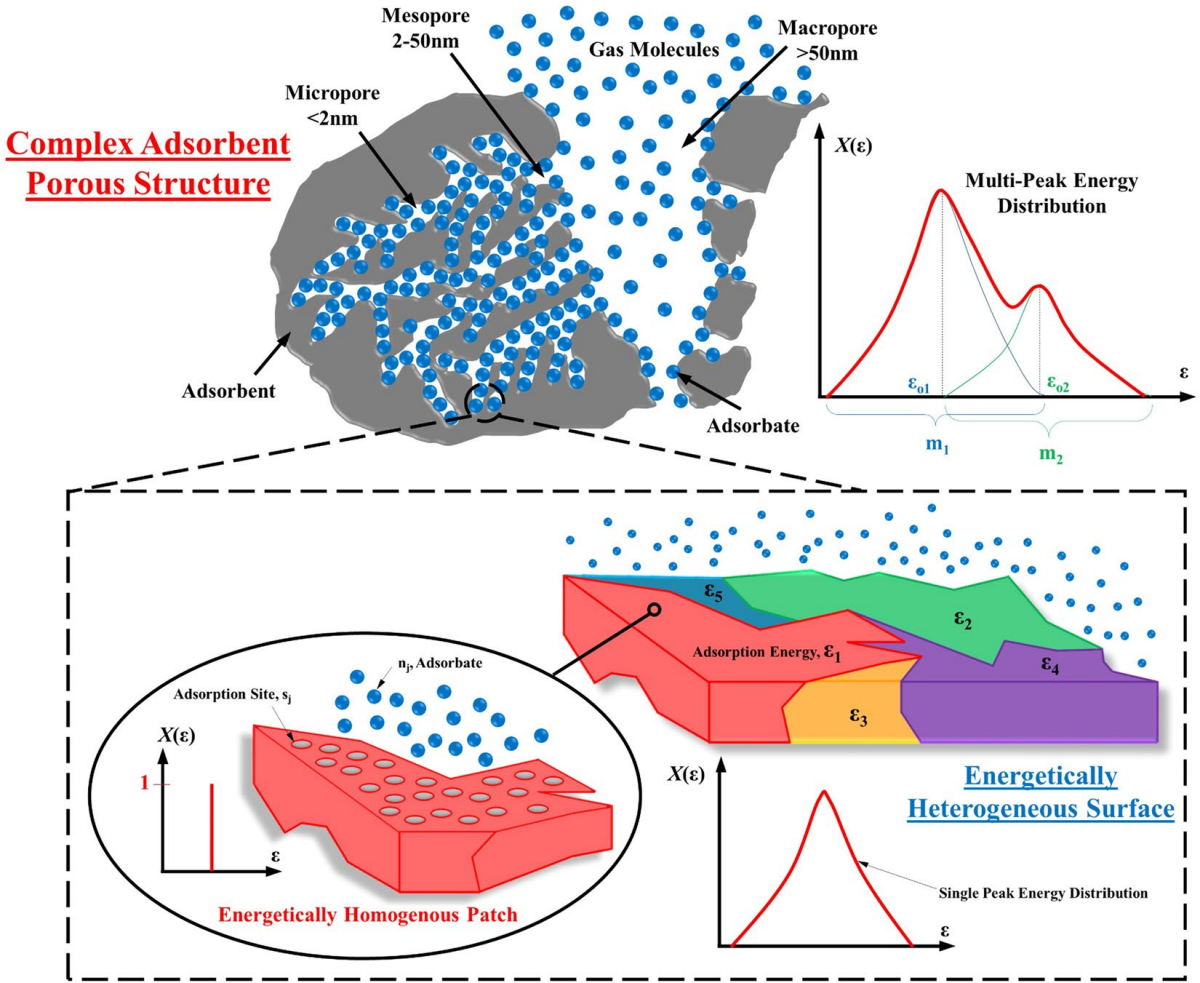
Adsorption on Complex Heterogeneous Porous Surface.

The understanding of sorption phenomena on porous adsorbents have many industrial and environmental applications, ranging from cooling, dehumidification, desalination, gas separation and storage. These processes improve not only our daily lives but also have the potential to lower carbon footprint by recovering low temperature waste heat sources for useful purposes. For example, separation of gas species for the selective removal of toxic pollutants from the air, desalination of seawater or highly impaired water, and the storage of Methane and Hydrogen onto suitable adsorbents etc., would depend on the efficacy and the long term reliability of the sorption processes. The surfaces of an adsorbent are complex by nature. In physical sorption, the adsorbate uptake is effected in the mono and/or multi-layer configurations where gas molecules are captured by the site energies of the porous adsorbents, which are categorized into three sizes of pores, namely macro-pores (>50 nm), meso-pores (2–50 nm) and micro-pores (<2 nm)^1^, as shown in Fig. 1. Such pores occur naturally or can be chemically/thermally functionalized to form the desired heterogeneous surfaces with prescribed level of energy sites of assorted probabilities for vapor capture, resulting in a useful shape or form of these isotherms. Such isotherms, most importantly, provide the design data needed for configuring the sorption-based process cycles. Based upon the shapes of the isotherms, the International Union of Pure and Applied Chemistry (IUPAC) has categorized all available adsorption isotherms into six types. Although, isotherm modeling has been reported extensively in the literature, however, it still lacks a universal approach in predicting the adsorption isotherms of all available types.

The conventional isotherm models of Henry, Tόth and Langmuir^2–4^, are only valid in the low pressure ranges i.e. Henry region with respect to the saturation conditions. Jaroniec and Marczewski^5–8^ followed a similar approach of Tόth, by using a power function^9^ but appeared imprecise in the transition from Henry to saturation region. Similarly, Dubinin-Astakhov (DA) and Dubinin-Radushkevich (DR) yield improved isotherm models that could perform well near saturation regions but are less consistent in the low pressure regions^10–13^. The incorporation of Fermi-Dirac distribution concept to the Langmuir model^14^ showed a significant consistency of isotherm predictions for Type-I to Type-III and Type-V, however, less successful in capturing the behavior of remaining isotherms i.e., Type-IV and Type-VI. In another approach using the concept of grand-canonical-ensemble in BET models^15^, the isotherm predictions have improved for Type-I to Type-V. The statistical mechanical formulation to isotherm model was introduced by Yahia *et al*.^16^ and he had successfully applied it to the multi-layer formation of Type-VI isotherms. However, none of the models reported hitherto were able to generalize the behavior of all isotherm types with a unified approach.

Ross and Olivier proposed the concept of Homotattic Patch Approximation (HPA)^17^ where the heterogeneous surface of adsorbent is sub-divided into finite homogeneous patches, each having a constituent site energy that forms the energy distribution functions over the surfaces. Frendlich^18^, Sips^19, 20^, Fowler^21^, and Jovanovich’s^22^ models showed good agreement with most of the isotherm types by adopting the HPA approach. Despite these conceptual improvements, the models still lack consistency in predicting all adsorption isotherms types^23^ as the heterogeneous adsorbent surfaces are more complex, comprising of the multiple sets of adsorption site energies and levels. Graham^24^ proposed a fractional parameter to be introduced to such set of site energy, treating each of them as separately. Tang *et al*.^25^ validated such an approach with simple Langmuir model for high pressure adsorption, however, the concept was limited to Type-I isotherm as expected. Despite the HPA and the multiple sets of site energy, that have given more insight to the complex adsorption phenomena, these approaches have not been unified yet to yield a universal model for all isotherm types. In this paper, a unified approach is considered for an adsorption isotherm model by combining the three concepts, namely (i) the HPA, (ii) the revised Langmuir model and (iii) the fractional probability factor for the distribution of site energy sets. In the later sections, the proposed universal model will be validated with Type-I to Type-VI isotherms available from the literature.

## Results and Discussion

### Type-I Adsorption Isotherm

**Figure 2:**
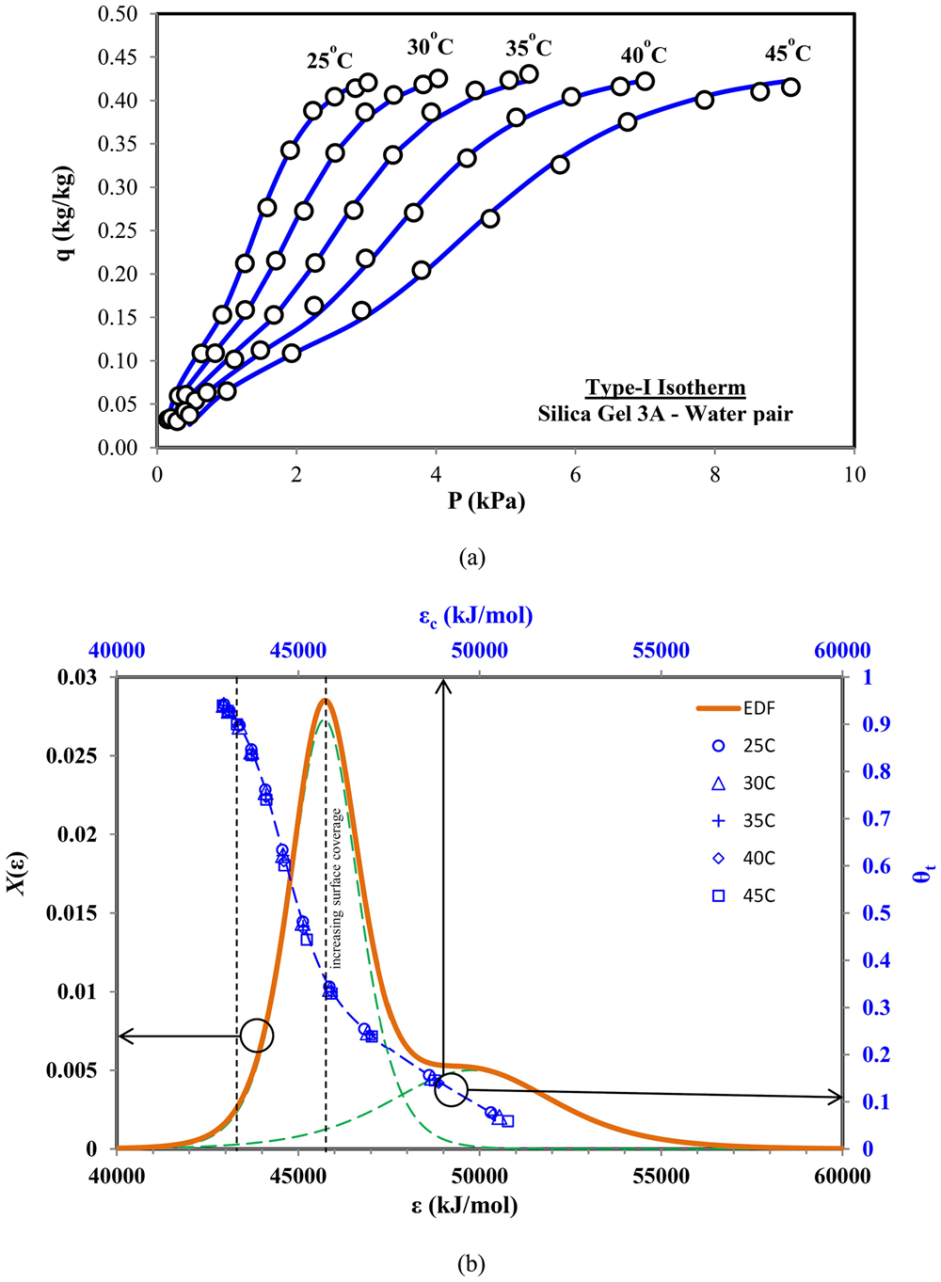
(**a**) Predicted and measured data for Type-I adsorption isotherms (Silica gel 3A-water pair) and (**b**) corresponding combined adsorption sites energy distribution.

The Type-I based adsorption isotherm is mostly characterized as a monolayer isotherm that can be fitted using simple Langmuir model. At the start, the Type-I isotherm shows a rapid increase in the adsorption uptake with increase in the pressure or concentration, followed by the gentle gradient in the uptake until the saturation pressure. Figure 2(a) shows the experimental data for Silica Gel 3A and water, adsorbent-adsorbate pair isotherms obtained at different temperatures and pressures^26^, categorized as the Type-I isotherms by IUPAC. These isotherms are validated using the proposed universal isotherm model with two-term representation of adsorption site energy sets. The circular dots in Fig. 2(a) denotes the experimental data points of the silica gel (Type 3A)-water pair isotherms whilst the blue line are the predictions from the universal isotherm model.

On closer examination of the isotherms, a small saturation step of adsorption uptake can be noted at the lower concentration regions. Beyond these steps, the adsorption uptake showed a rapid rise and they leveled-off asymptotically towards the saturation limit. Instead of single set of adsorption sites, as typified by the Langmuir model for Type-I isotherms. Figure 2(b) illustrates the dual probability peaks of energy distribution function (EDF), representing the availability of two distinct sets of adsorption energy sites. Firstly, during the initial uptake at low concentrations, only high energy adsorption sites with low probability peak are available for the adsorbent molecules, due to high value of threshold site energy *ε*_*c*_. Conversely, at higher concentrations, lower adsorption energy sites with high availability (or probability peak of EDF) leads to a rapid increase in the surface coverage and contributes to the major adsorbate uptake. The cumulative uptake or surface coverage (*θ*_*t*_) is presented on the secondary axis of Fig. 2(b), against corresponding *ε*_*c*_ value. The levelling of adsorption uptake occurs as the probability value approaches to zero, signifying the saturation of adsorption sites.

Depending upon the site energy value with peak probability of the energy distribution function, for different sets of adsorption energy sites, it can be determined whether the higher adsorption uptake can be observed at lower concentration or higher. If there is less availability of higher adsorption energy sites and their probability peak is closer to the probability peak of the lower adsorption energy sites, then a gradual increase in the adsorption uptake toward saturation can be observed, as considered by the simple Langmuir model for Type-I isotherm, without any intermediate saturation.

### Type-II Adsorption Isotherm

**Figure 3:**
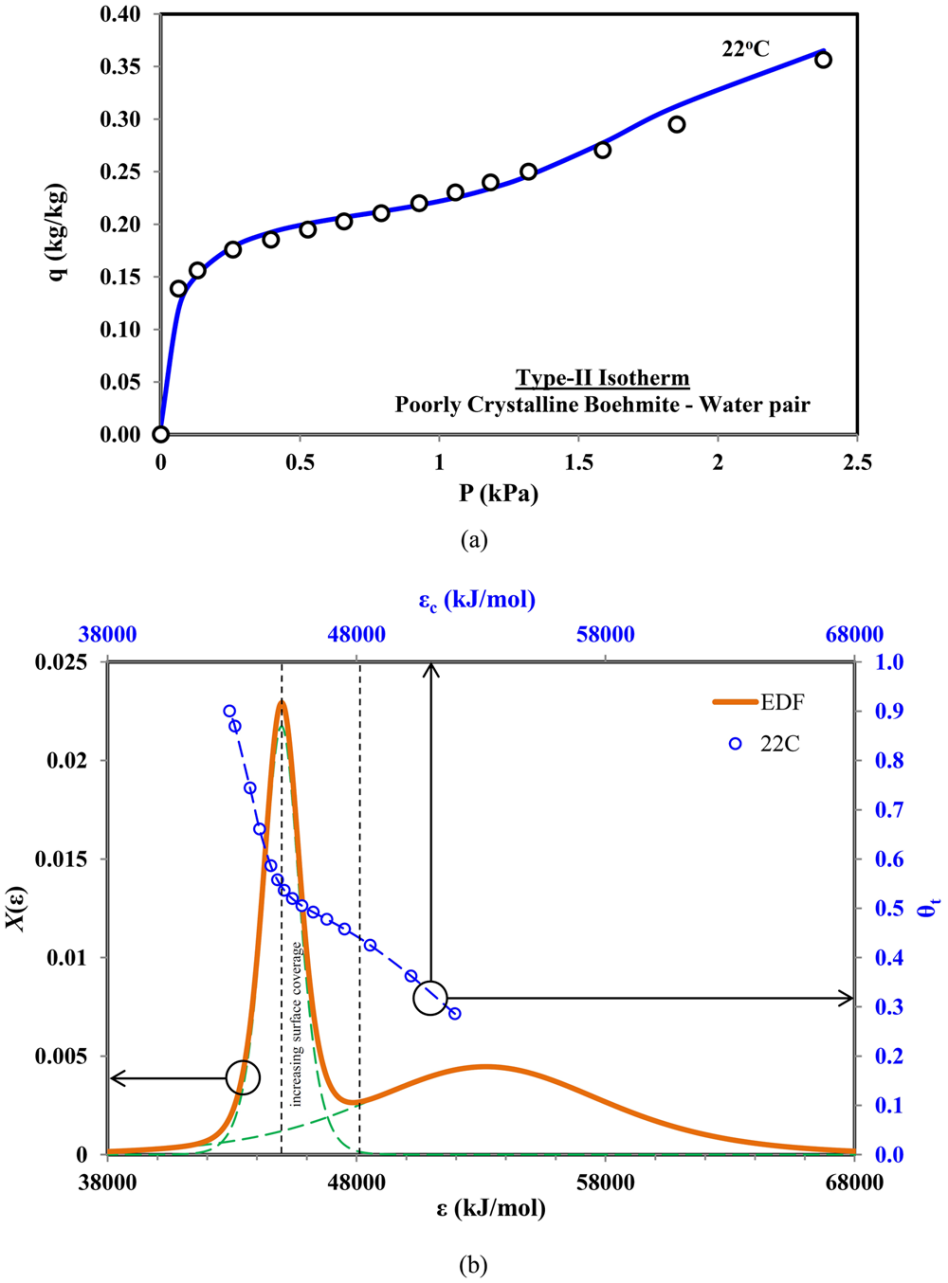
(**a**) Predicted and measured data for Type-II adsorption isotherms (Poorly crystalline Boehmite-water pair) and (**b**) corresponding combined adsorption sites energy distribution.

Type II isotherms are almost similar in shape to Type-I except that the uptake trend is continuous with increase in the pressure ratios without any phenomena of ‘pleateau’ even when the relative pressure approaching unity. Such differences in trends are attributed to availability of assorted pore sizes (macro, meso and micro pores) on the adsorption surfaces. Considering the experimental data of the poorly-crystalline boehmite and water pair isotherm^27^ in Fig. 3(a), which is predicted using proposed universal adsorption isotherm model with two terms, representing two different pore size groups. An excellent prediction for both the experimental data and its trend is obtained by the proposed universal model.

In order to further investigate the adsorption uptake against the availability of adsorption sites, the energy distribution graph and the adsorption uptake against the adsorption energy sites are shown in Fig. 3(b). The energy distribution graph shows dual peak pattern; one with smaller probability values but with larger span across higher energy sites, and the other depicts a much higher peak probability values but it has a smaller variance at the lower energy sites. The model predicts a rapid increase in the uptake at lower energy levels with higher probability. However, it was increasing gradually for high energy sites with lower probability. Although the energy distribution pattern is similar to the Type-I isotherms, the uptake is higher at lower pressure/concentrations due to significant contributions by the second group of pore size with high adsorption energy sites. The model indicates that both groups of energy sites or pore sizes contribute almost equally to the adsorption uptake, as evident by the values of *α*_1_ and *α*_2_ which are 0.4265 and 0.5735 respectively.

### Type-III Adsorption Isotherm

**Figure 4:**
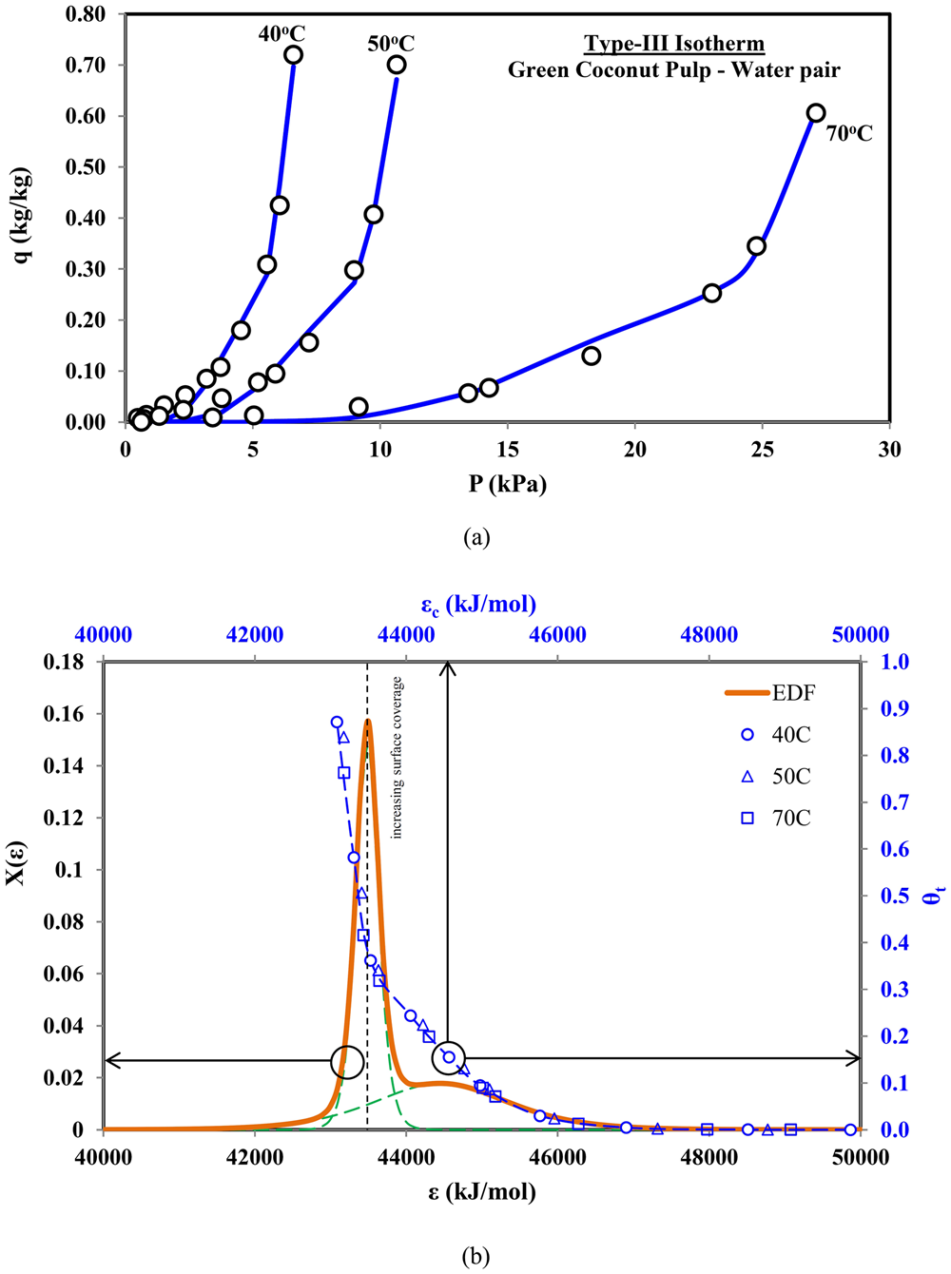
(**a**) Predicted and measured data for Type-III adsorption isotherms (Green coconut pulp-water pair) and (**b**) corresponding combined adsorption sites energy distribution.

For Type-III isotherms, the adsorbate uptake increases exponentially with increasing pressure/concentration, as shown in Fig. 4(a), and the increase continues until the relative pressure reaches unity. Such Type-III isotherms are obtained from adsorbent-adsorbate pair of green coconut pulp and water^28^. Based upon the universal model with two terms, the predictions are shown by the blue lines in Fig. 4(a). The energy distribution of the adsorption site energy along with the adsorption uptake is also shown in Fig. 4(b).

Similar to Type-I and Type-II isotherms, the energy distribution function has a dual peak but they are clustered closer with overlapping energy levels in the abscissa. With the availability of high energy level adsorption sites, a gradual increase in the adsorption uptake can be seen with increase in the probability value, Fig. 4(b). After peak probability value of high energy sites, the other set of lower adsorption energy sites become available, causing further but sharp increase in the adsorption uptake, due to their high availability/probability. The uptake increases rapidly after the energy site of peak probability, until it reaches the limit of saturation pressure.

### Type-IV Adsorption Isotherm

**Figure 5:**
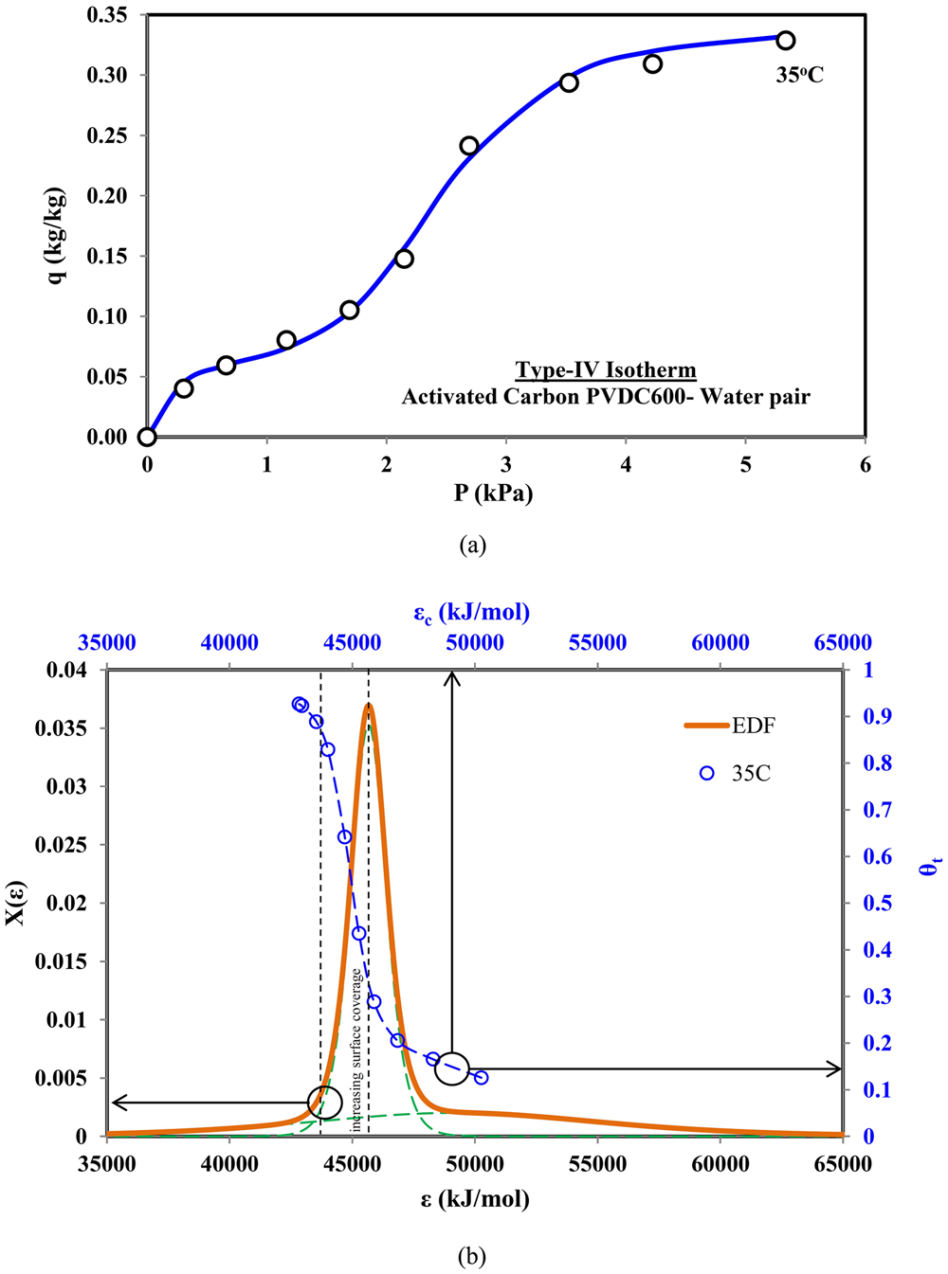
(**a**) Predicted and measured data for Type-IV adsorption isotherms (Activated carbon PVDC600-water pair) and (**b**) corresponding combined adsorption sites energy distribution.

The Type-IV isotherm is the characteristics of both Type-II and Type-I isotherms. For example, there is a prominent intermediate saturation of adsorption surface at the low pressure/concentration. However, at higher concentration/pressure, the adsorption uptake again experiences saturation near the saturation pressure. This kind of behavior exhibits the multi-layer formation during adsorption. The Type-IV isotherm behavior is shown by the activated carbon and water pair, for which the experimental data is obtained from^29^, and the isotherm prediction is made with universal adsorption model, as shown in Fig. 5(a). The predictions are depicted with two-term universal adsorption model where the energy distribution function, given by Fig. 5(b), has the appearance of a non-symmetrical single peak, a unique feature of the Type-IV isotherm. This unique EDF depicts the significant contribution from the multi-layer formation as the effect from the second site energy probability peak diminishes. The variation of adsorption uptake against the adsorption energy sites, is similar to the Type-I isotherm with rapid increase in the uptake after crossing the peak probability energy site, except there is a prominent horizontal saturation at the start, demonstrating multi-layer formation. It then again approaches to horizontal saturation when the probability of adsorption energy sites reaches zero.

### Type-V Adsorption Isotherm

**Figure 6:**
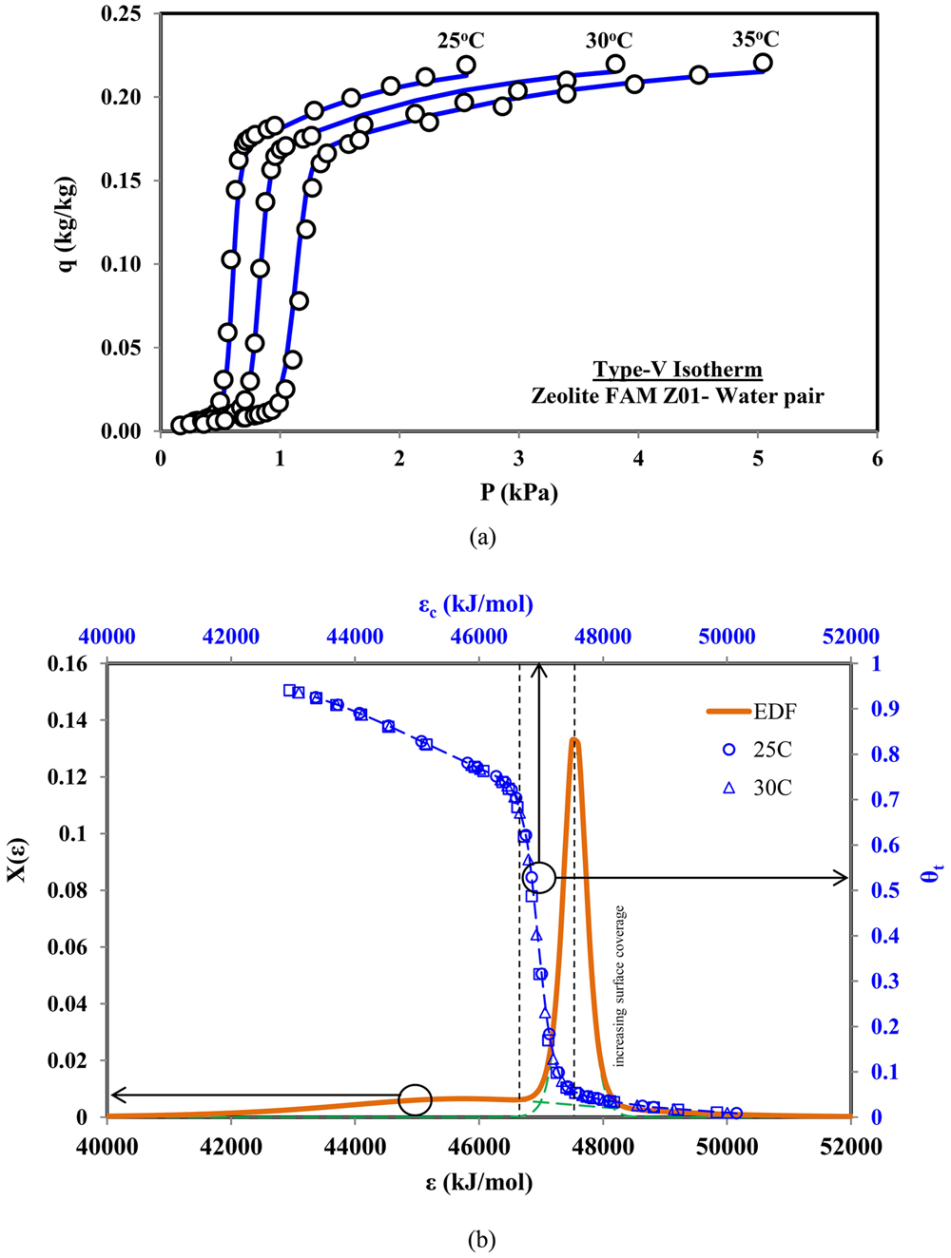
(**a**) Predicted and measured data for Type-V adsorption isotherms (Zeolite FAM Z01-water pair) and (**b**) corresponding combined adsorption sites energy distribution.

The Type-V isotherms have the ‘*S’* shape characteristic, comprising three distinctive uptake rates. At low concentrations, the uptake rate is gentle and then a rapid rise at intermediate concentrations is observed, which then level-off at higher concentrations, as shown in Fig. 6(a). Such Type-V isotherms are found in the Zeolite Z01 and water pair^26^, which can be equally predicted by the two-term universal isotherm model. The notable difference between the Type-V and the previous types of isotherms is the higher probability peak of the energy distribution curve, occurring at low energy sites instead of at higher energy sites, as shown in Fig. 6(b). Consequently, higher surface coverage is observed even at lower concentrations.

### Type-VI Adsorption Isotherm

The Type-VI isotherm is considerably more complex and a special category in the shapes of isotherms, comprising two or more patterns of Type IV in multi-layer formation, showing adsorption of adsorbate molecules up to the limit of condensation phenomena. Such Type-VI isotherms can be found in the adsorption of methane (CH_4_) over the porous surface of MgO, at a cryogenic temperature of 87.4 K or −185.75 °C^30^. For the isotherm prediction, the improvised universal isotherm model with four-terms representing each of the adsorption layers formed on the heterogeneous surfaces. However, the unity limit for the probability factors of adsorption energy sites sets is still perceived i.e. *α*_1_ + *α*_2_ + *α*_3_ + *α*_4_ = 1.

**Figure 7:**
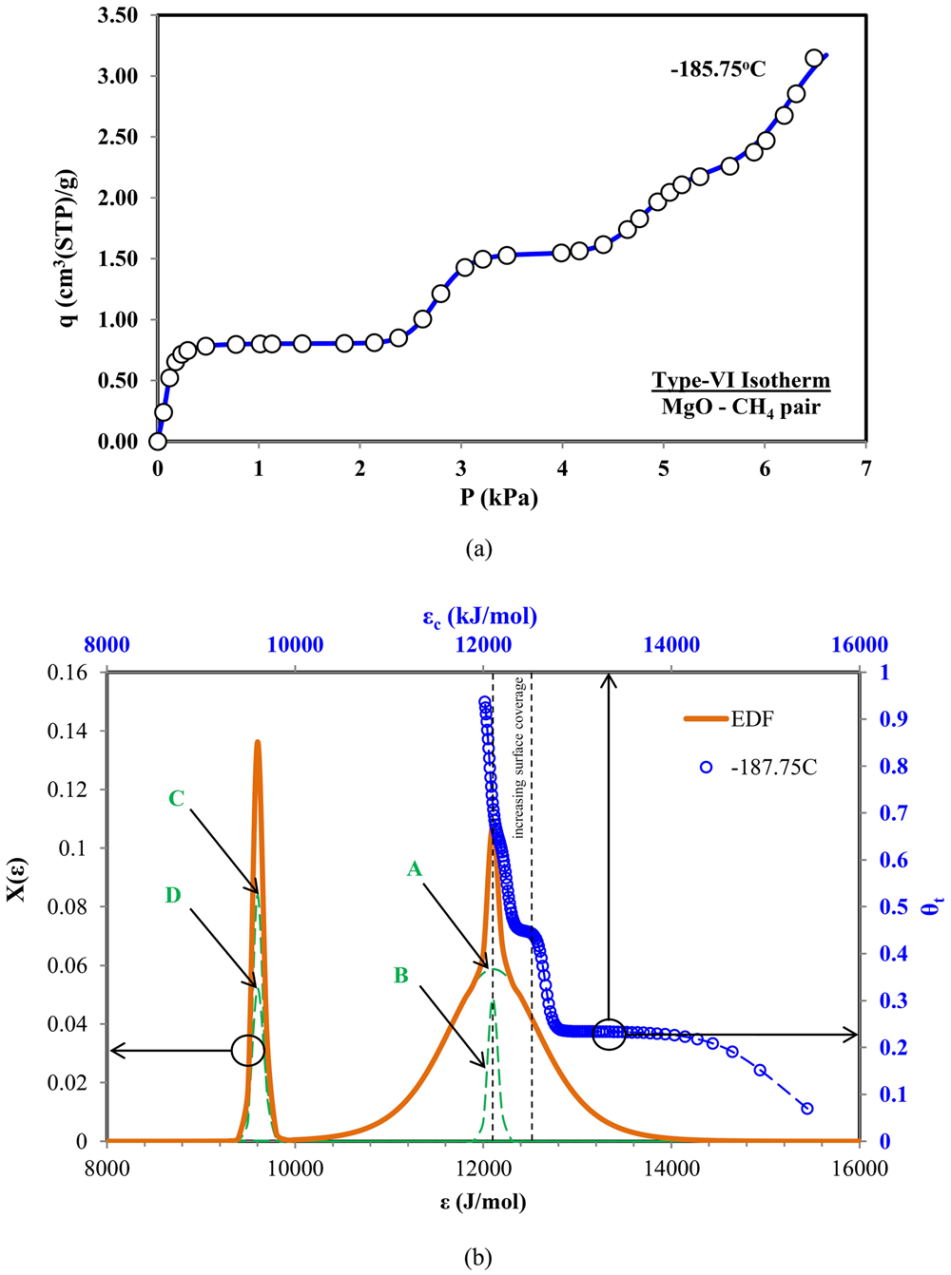
(**a**) Predicted and measured data for Type-VI adsorption isotherms (MgO-CH_4_ pair) and (**b**) corresponding combined adsorption sites energy distribution.

The experimental data for Type-VI isotherm and its prediction using the universal adsorption isotherm model are shown in Fig. 7(a). The notable characteristics of Type VI isotherms, as in this example, is captured uniquely by the four-peak energy distributions of separate site energy in an unique arrangement. One pair of constituent energy distributions (A and B) coincidently have the site energy, *ε*, but each energy distribution has a separate probability level and a significantly different energy spread or variance. The other energy distribution pair (C and D) have a lower site energy with a narrower but identical variances as compared to the previous pair. Consequently, their combined energy distribution energy resulted in an apparent “two-peak” energy distribution, as shown by the green-line of Fig. 7(b). Such a combined energy distribution is frequently misinterpreted as having only two constituent energy distribution function rather than the four-peak. Also observed in Fig. 7(b), the coverage versus threshold site energy (*ε*_*c*_) plot indicates almost no contribution from the second pair of having lower energy site. This can be attributed to the *ε*_*c*_ values which are higher than the site energy values of C and D distribution. Hence, the condensation approximation rule excluded any contribution to the coverage at these lower energy sites (as compared with *ε*_*c*_).

From the above validation of the universal isotherm model with all six types of isotherms, the multiple site energy distribution function has inextricable link to the thermodynamics of the isotherms over a porous heterogeneous surface. This theoretical insight, between the surface coverage behaviour with respect to the adsorption energy site availability, offers great opportunities for material scientists to configure or tailor a purpose-built vapor uptake pattern. The surface energy sites onto the heterogeneous adsorbent could be formulated^31–33^, targeting applications either to improve the efficiency of existing sorption-driven processes or designing innovative hybrid processes to utilize low grade waste heat sources.

## Conclusion

Based upon the approach of energy distribution functions and homotattic patch approximation, the efficacy of universal adsorption isotherm model has been demonstrated in capturing the isotherms of all types of adsorbent-adsorbate pairs categorized by the IUPAC. The integral insight demonstrating the inextricable link between the mono and multi-layer formation of adsorbate with respect to the multi-peak energy distribution functions has enabled the unification of all isotherm models available hitherto. For isotherm Type-I to Type-V, it has been observed that the dual peaks of energy distribution with varied probability values and their alternate arrangements toward higher or lower energy sites, generate a variety of isotherms of the heterogeneous adsorbents. Only Type VI isotherms requires four-term model due to availability of multiple sets of adsorption energy sites and multi-layer formation. The universal isotherm model is deemed novel at this juncture, tracing characteristics of complex porous heterogeneous surface and potentially, its application towards configuring new adsorbent-adsorbate pairs with improved isotherms is boundless.

## Materials and Methods

### Universal Adsorption Isotherm Model

For modelling purposes, the heterogeneous surfaces of adsorbent is approximated with an agglomeration of numerous HPA homogenous patches where homogeneity is assumed in each site in terms of same energy level. The size of homogeneous patches is defined in terms of the energy distribution function (EDF) of adsorption site energy: The higher probability of an adsorption site will dictate a higher localized adsorption uptake within a mono-layer coverage. However, such an energy distribution may vary according to the pore types. The same concept is also applicable to the multi-layer formation. To accommodate for such variations, a fractional probability factor is introduced to define the fraction of the total surface coverage or the energy distribution, associated with each pore size or adsorbate layers of multi-layer formation.

By assuming one molecule per adsorption site and the homotattic patch approximation (CPA), the local adsorption uptake at such a homogeneous patch is given by;

1 $$\theta {(\varepsilon )}_{j}=\frac{{n}_{j}}{{s}_{j}}$$

where *n*_*j*_ denotes the number of molecules and *s*_*j*_ denotes the available local adsorption sites. Hence, the total adsorption uptake available at an adsorbent surface can be written as

2 $${\theta }_{t}=\frac{{N}_{a}}{{S}_{o}}=\sum _{j=1}^{\infty }\theta {(\varepsilon )}_{j}$$

where *N*_*a*_ and *S*_*o*_ represent the total number of adsorbed molecules and sites respectively. Equation (2) can be expanded as a sum of all local coverage and the fractional availability of respective adsorption sites over total surface i.e. ${s}_{i}/{S}_{o}$.

3 $${\theta }_{t}=\frac{{s}_{1}}{{S}_{o}}{\theta }_{1}+\frac{{s}_{2}}{{S}_{o}}{\theta }_{2}+\frac{{s}_{3}}{{S}_{o}}{\theta }_{3}+\mathrm{........}+\frac{{s}_{\infty }}{{S}_{o}}{\theta }_{\infty }$$

The fractional factors are proportional to the energy distribution function *X(ε)*, and the summation of these factors will be equal to unity, over the entire adsorption surface, i.e.,

4 $$\frac{{s}_{i}}{{S}_{o}}(\varepsilon )=X(\varepsilon )d\varepsilon $$

5 $$\mathop{{\int }_{0}^{\infty }X(\varepsilon )d\varepsilon }\limits_{\mathop{\longleftrightarrow }\limits_{all\,sites}}=\sum _{n=1}^{\infty }\frac{{s}_{i}}{{S}_{o}}(\varepsilon )=1$$

From mathematics, the total adsorption uptake or coverage of the heterogeneous surface with quasi-static patches, can be expressed by the integration given by equation (6).

6 $${\theta }_{t}=\mathop{{\int }_{0}^{\infty }\{\theta (\varepsilon )X(\varepsilon )\}d\varepsilon }\limits_{\mathop{\longleftrightarrow }\limits_{all\,sites}}$$

Hence, equation (6) estimates the total coverage of an adsorbent surface when the localized adsorption uptake *θ(ε)* and the energy distribution function *X(ε)*, are known. By knowing the rate of adsorption and desorption, the rate of adsorption uptake can be given by equation (7).

7 $$\frac{d\theta (\varepsilon )}{dt}={R}_{a}-{R}_{d}$$

where R_a_ and R_d_ are the rate of adsorption and desorption respectively, given by the classical Absolute Rate Theory (ART)^34, 35^.

8 $$\frac{d\theta (\varepsilon )}{dt}={K}_{a}\,p(1-\theta )\exp (\frac{-{\varepsilon }_{a}}{RT})-{K}_{d}\theta \exp (\frac{-{\varepsilon }_{d}}{RT})$$

However, in equilibrium, the rate of adsorption and desorption become equal i.e. $\frac{d\theta (\varepsilon )}{dt}=0$. So, by letting $K={K}_{a}/{K}_{d}$ and Δ*ε* = *ε*_*d*_ − *ε*_*a*_ = *ε* − *h*_*fg*_, equation (8) can be simplified to the revised Langmuir isotherm equation, as equation (9).

9 $$\theta (\varepsilon )=\frac{Kp\,\exp (\frac{{\rm{\Delta }}\varepsilon }{RT})}{(1+Kp\,\exp (\frac{{\rm{\Delta }}\varepsilon }{RT}))}$$

From ${\varepsilon }_{c}=-RT\,\mathrm{ln}\,Kp$, the Langmuir isotherm equation (9) can be modified as:

10 $$\theta (\varepsilon )=\frac{\exp (\frac{{\rm{\Delta }}\varepsilon -{\varepsilon }_{c}}{RT})}{(1+\exp (\frac{{\rm{\Delta }}\varepsilon -{\varepsilon }_{c}}{RT}))}$$

The equation (10) shows the temperature dependence of the adsorption uptake *θ(ε)*. By invoking the condensation approximation (CA), the equation (10) can be decomposed into two parts and can be simplified as equation (11).

11 $$\begin{array}{c}{\theta }_{t}={\int }_{0}^{{\varepsilon }_{c}}(0\times X(\varepsilon ))d\varepsilon +{\int }_{{\varepsilon }_{c}}^{\infty }(1\times X(\varepsilon ))d\varepsilon \,CA:[\mathop{\mathrm{lim}}\limits_{T\to 0}\theta (\varepsilon )={\theta }_{c}(\varepsilon )=\{\begin{array}{c}0\,for\,{\rm{\Delta }}\varepsilon \le {\varepsilon }_{c}\\ 1\,for\,{\rm{\Delta }}\varepsilon \ge {\varepsilon }_{c}\end{array}]\\ {\theta }_{t}={\int }_{{\varepsilon }_{c}}^{\infty }X(\varepsilon )d\varepsilon \end{array}$$

The equation (11) shows that the total adsorption uptake (*θ*_*t*_) is only depending upon the energy distribution function of adsorption energy sites, *X(ε)*. Therefore, the key to accurately capture the adsorption uptake is the correct prediction of adsorption sites distribution over the surface of multiple heterogeneous patches. To capture the adsorbent surface heterogeneity, although the multi-peaks representation of energy distribution have been reported widely in the literature^23^, there is yet a unified mathematical expression that can accurately capture and quantify the individual energy distribution function to the combined distribution function. For this purpose of fractional representation to the total adsorption uptake between micro and macro pores of an adsorbent or among multi-layer formations, we introduce a mathematical form of symmetrical Gaussian function which is embedded with probability factor *α*, to meaningfully capture the characteristics of the energy distribution of adsorption sites over the heterogeneous surface i.e.

12 $$X(\varepsilon )=\sum _{i=1}^{n}{\alpha }_{i}\,{\{\frac{\exp (\frac{{\rm{\Delta }}\varepsilon -{\varepsilon }_{oi}}{{m}_{i}})}{{m}_{i}{[1+\exp (\frac{{\rm{\Delta }}\varepsilon -{\varepsilon }_{oi}}{{m}_{i}})]}^{2}}\}}_{i}$$

where *ε*_*oi*_ represents the adsorption energy site with maximum frequency, which also predicates the relative position or mean value of the energy distribution curve along the energy axis. The factor *m*_*i*_ mathematically depicts the overall spread or the standard deviation of the energy distribution curve from its mean value, *ε*_*oi*_. However, in physical sense, *m*_*i*_ represents the surface heterogeneity or the range of the energy sites available for the adsorption. As *ε*_*oi*_ and *m*_*i*_ represent mean and deviation of an energy term, respectively therefore they have the same units as the energy term. On the other hand, the sum of all of the probability parameters *α*_*i*_ is equal to ‘1’ i.e. $\sum _{i=1}^{n}{\alpha }_{i}=1$.

The equation (12) provides an insight to the surface characteristics such as pore opening, surface area and pore size distributions as these parameters are modified or functionalized. Hence, a dedicated porous adsorbent of desired energy distributions can now be configured by a chemist or material scientist. The mean and the variance/deviation of surface energy levels of materials can be tailored with surface treatment techniques such as acidification^36^, chemical^37^ and heat treatment^38–41^, to configure the surface energy distributions i.e. altering the pore sizes of adsorbent to achieve a desired isotherm shape^42, 43^. The energy distribution function is integrated over the available adsorption energy site to get the overall adsorption uptake;

13 $${\theta }_{t}={\int }_{{\varepsilon }_{c}}^{\infty }\,[\sum _{i=1}^{n}{\alpha }_{i}\,{\{\frac{\exp (\frac{{\rm{\Delta }}\varepsilon -{\varepsilon }_{o}}{m})}{m{[1+\exp (\frac{{\rm{\Delta }}\varepsilon -{\varepsilon }_{o}}{m})]}^{2}}\}}_{i}]d\varepsilon $$

By solving the integral and letting ${\varepsilon }_{c}=-RT\,\mathrm{ln}\,Kp$, a more concise expression is obtained i.e.

14 $${\theta }_{t}=\sum _{i=1}^{n}{\alpha }_{i}\,{\{{[1+\exp (\frac{-RT\mathrm{ln}Kp-{\varepsilon }_{o}}{m})]}^{-1}\}}_{i}$$

Upon further simplification and by letting the adsorption equilibrium constant $K=1/{p}_{s}$, where *p*_*s*_ represents the saturation pressure at the maximum possible uptake by an adsorbent, the universal adsorption isotherm model is obtained as:

15 $${\theta }_{t}=\sum _{i=1}^{n}{\alpha }_{i}\,{\{\frac{{(\frac{p}{{p}_{s}}\exp (\frac{{\varepsilon }_{oi}}{RT}))}^{\frac{RT}{mi}}}{1+{(\frac{p}{{p}_{s}}\exp (\frac{{\varepsilon }_{oi}}{RT}))}^{\frac{RT}{mi}}}\}}_{i}$$

It is important to mentioned here that the larger value of *m* depicts high surface heterogeneity, resulting in the smaller slope of the isotherm graph and vice versa, due to wider spread and low fractional peak of the corresponding energy distribution curve.

For Type-I to Type-V, the energy distribution function is validated to have two nominal peaks, i.e., *n* = 2. Therefore, the equation (15) can be decomposed with two probability factors, i.e., *α*_1_ and *α*_2_, where *α*_1_ + *α*_2_ = 1.

16 $${\theta }_{t}={\alpha }_{1}\,[\frac{{(\frac{p}{{p}_{s}}\exp (\frac{{\varepsilon }_{o1}}{RT}))}^{\frac{RT}{m1}}}{1+{(\frac{p}{{p}_{s}}\exp (\frac{{\varepsilon }_{o1}}{RT}))}^{\frac{RT}{m1}}}]+{\alpha }_{2}\,[\frac{{(\frac{p}{{p}_{s}}\exp (\frac{{\varepsilon }_{o2}}{RT}))}^{\frac{RT}{m2}}}{1+{(\frac{p}{{p}_{s}}\exp (\frac{{\varepsilon }_{o2}}{RT}))}^{\frac{RT}{m2}}}]$$

Similarly, for the Type-VI Isotherm, the corresponding energy distribution functions is demonstrated with four probability factors, i.e., *α*_1_ *, α*_2_ *, α*_3_ and *α*_4_, due mainly to the four-peak constituent energy distributions where *α*_1_ + *α*_2_ + *α*_3_ + *α*_4_ = 1. The corresponding decomposed form is given as;

17 $$\begin{array}{c}{\theta }_{t}={\alpha }_{1}\,[\frac{{(\frac{p}{{p}_{s}}\exp (\frac{{\varepsilon }_{o1}}{RT}))}^{\frac{RT}{m1}}}{1+{(\frac{p}{{p}_{s}}\exp (\frac{{\varepsilon }_{o1}}{RT}))}^{\frac{RT}{m1}}}]+{\alpha }_{2}\,[\frac{{(\frac{p}{{p}_{s}}\exp (\frac{{\varepsilon }_{o2}}{RT}))}^{\frac{RT}{m2}}}{1+{(\frac{p}{{p}_{s}}\exp (\frac{{\varepsilon }_{o2}}{RT}))}^{\frac{RT}{m2}}}]\\ \quad \quad +{\alpha }_{3}\,[\frac{{(\frac{p}{{p}_{s}}\exp (\frac{{\varepsilon }_{o3}}{RT}))}^{\frac{RT}{m3}}}{1+{(\frac{p}{{p}_{s}}\exp (\frac{{\varepsilon }_{o3}}{RT}))}^{\frac{RT}{m3}}}]+{\alpha }_{4}\,[\frac{{(\frac{p}{{p}_{s}}\exp (\frac{{\varepsilon }_{o4}}{RT}))}^{\frac{RT}{m4}}}{1+{(\frac{p}{{p}_{s}}\exp (\frac{{\varepsilon }_{o4}}{RT}))}^{\frac{RT}{m4}}}]\end{array}$$

It must be noted that the Equations (16) and (17) are the expanded forms of equation (15) according to the availability of the sets of adsorption sites and their distribution in an isotherm, represented by the probability factor *α*_*i*_. However, the total number of expanded terms required in the model, depends upon the need to fully capture the behavior of an isotherm type. For Type-I to Type-V isotherms, only two terms (i.e. *n* = 2) are enough to fully predict their behavior. However, for Type-VI isotherm, equation (15) is utilized in four term form (i.e. *n* = 4) to fully predict its complex multi-layer behavior, thereby increasing the total number of corresponding model parameters i.e. *ε*_*oi*_, *m*_*i*_ and *α*_*i*_. The total number of the expanded terms for Type-VI isotherm can be less or more than four, depending upon its complexity in form of multi-layer behavior. More complex characteristics need more terms, thereby increasing number of model parameter and computational effort/time, depicting model limitation for Type-VI isotherm only. However, from performance prediction point of view, the proposed universal adsorption isotherm model has no limitation as it captures the behavior of all six isotherm types with accuracy.

The applicability of the universal adsorption isotherm model is validated using the behavior of all six types of the IUPAC isotherms, as reported in the literature. For each type of the adsorbent-adsorbate pair, the values of the adsorbent site energies at maximum probability (*ε*_*oi*_), the surface heterogeneity parameter or spread (*m*) of energy distribution functions (EDFs), and the fractional probability factors (α_i_) of a constituent EDF for site energy set, can be estimated. Using these parameters, the universal model predicts the isotherms or vapor uptake of the adsorbent-adsorbate pair. The detailed parameters of universal adsorption isotherm model are presented in the results and discussion section for either the two-term model (Eq. 16) or the four-term model (Eq. 17), to give the corresponding coverages of adsorbate uptake of all isotherms.

### Data Availability

All data generated or analysed during this study are included in this published article (and its Supplementary Information).

## Electronic supplementary material

Supplementary Information
