# Supplementary material for: A Universal Isotherm Model to Capture Adsorption Uptake and Energy Distribution of Porous Heterogeneous Surface
Source: Sci Rep. 2017 Sep 6;7:10634. doi: 10.1038/s41598-017-11156-6 (PMC5587542; doi:10.1038/s41598-017-11156-6)
Supplement: Supplementary file 1 — Supplementary Information [file 41598_2017_11156_MOESM1_ESM.pdf]

A Universal Isotherm Model to Capture Adsorption Uptake and Energy Distribution of Porous Heterogeneous Surface

[1]Kim Choon Ng, [2]Muhammad Burhan, [3]Muhammad Wakil Shahzad, [4]Azahar Bin Ismail

[1-4]King Abdullah University of Science and Technology, Thuwal 23955-6900, Saudi Arabia.

Emails: [1]kimchoon.ng@kaust.edu.sa, [2]muhammad.burhan@kaust.edu.sa, [3]muhammad.shahzad@kaust.edu.sa

Supplementary Information:

The parameters related to universal adsorption model for presented isotherm predictions are given in the tables below:

Table 1: Key values of parameters of universal adsorption model for Type-I isotherm

Parameter	Value	Parameter	Value
ε_{o1} (J/mol)	5799.9	ε_{o2} (J/mol)	1729.6
m1 (J/mol)	1529.9	m2 (J/mol)	635.2
α_1	0.307	α_2	0.693
P_s (kPa)	3.169	q*(kg/kg)	0.45

Table 2: Key values of parameters of universal adsorption model for Type-II isotherm

Parameter	Value	Parameter	Value
ε_{o1} (J/mol)	866.8	ε_{o2} (J/mol)	9078.4
m1 (J/mol)	490.4	m2 (J/mol)	3212.5
α_1	0.4265	α_2	0.5735
P_s (kPa)	2.645	q*(kg/kg)	0.42

Table 3: Key values of parameters of universal adsorption model for Type-III isotherm

Parameter	Value	Parameter	Value
ε_{o1} (J/mol)	477.75	ε_{o2} (J/mol)	1453.13
m1 (J/mol)	97.64	m2 (J/mol)	591.17
α_1	0.5789	α_2	0.4211
P_s (kPa)	4.247	q*(kg/kg)	0.8

Table 4: Key values of parameters of universal adsorption model for Type-IV isotherm

Parameter	Value	Parameter	Value
ε_{o1} (J/mol)	2096.47	ε_{o2} (J/mol)	5613.18
m1 (J/mol)	478.42	m2 (J/mol)	4025.90
α_1	0.6755	α_2	0.3245
P_s (kPa)	5.629	q*(kg/kg)	0.36

Table 5: Key values of parameters of universal adsorption model for Type-V isotherm

**Parameter**	**Value**	**Parameter**	**Value**
ε_{o1} (J/mol)	2309.31	ε_{o2} (J/mol)	4112.51
m1 (J/mol)	1352.90	m2 (J/mol)	121.31
α_1	0.3531	α_2	0.6469
P_s (kPa)	3.1699	q*(kg/kg)	0.23

Table 6: Key values of parameters of universal adsorption model for Type-VI isotherm

**Parameter**	**Value**	**Parameter**	**Value**
ε_{o1} (J/mol)	3133.83	ε_{o3} (J/mol)	235.07
m1 (J/mol)	342.77	m3 (J/mol)	32.32
α_1	0.2349	α_3	0.1978
ε_{o2} (J/mol)	640.15	ε_{o4} (J/mol)	45
m2 (J/mol)	39.58	m4 (J/mol)	29.37
α_2	0.2141	α_4	0.3532
P_s (kPa)	3.17	q*(kg/kg)	0.23
